# There is a need for a paradigm shift in laparoscopic surgical training: results of a nationwide survey among teaching hospitals in Switzerland

**DOI:** 10.1186/s12909-024-05209-4

**Published:** 2024-02-27

**Authors:** Karolina Wczysla, Moritz Sparn, Bruno Schmied, Dieter Hahnloser, Stephan Bischofberger

**Affiliations:** 1 Department of Surgery, Kantonsspital St. Gallen, Rorschacher Strasse 95, CH-9007 St. Gallen, Switzerland; 2 Department of Surgery, Centre Hôpitalier Universitaire Vaudois, CH-1011 Lausanne, Switzerland

**Keywords:** Surgical curriculum, Surgical training, Simulation in surgery, Proficiency-based surgical training, Laparoscopic training

## Abstract

**Background:**

Surgical training curricula have changed little over the past decades. Current advances in surgical techniques, especially in minimally invasive surgery, as well as the rapidly changing socioeconomic environment pose a major challenge for the training of young surgeons. The aim of this survey was to provide a representative overview of the surgical training landscape in Switzerland focusing on laparoscopic surgical training: How do department chairs of teaching hospitals deal with the above challenges, and what should a future training curriculum look like?

**Methods:**

This is a prospective, questionnaire-based, cross-sectional study among the heads of departments of all certified surgical teaching hospitals in Switzerland.

**Results:**

The overall response rate was 56% (48/86) and 86% (19/22) for tertiary centers. Two-thirds of the centers (32) organize themselves in training networks. Laparoscopic training courses are offered in 25 (52%) hospitals, mainly in tertiary centers. Self-training opportunities exist in 40 (83%) hospitals. In addition to commercial (27) and self-built (7) box trainers, high-fidelity trainers are available in 16 (33%) hospitals. A mandatory training curriculum exists in 7 (15%) facilities, and a training assessment is performed in 15 (31%) institutions. Thirty-two (65%) heads of departments indicated that residents have sufficient practical exposure in the operating room, but the ability to work independently with obtaining the specialist title is seen critically (71%). They state that the surgical catalog does not adequately reflect the manual skills of the resident (64%). The desire is for training to be restructured from a numbers-based to a performance-based curriculum (53%) and for tools to assess residents' manual skills (56%) to be introduced.

**Conclusions:**

Department chairs stated that the existing curriculum in Switzerland does not meet the requirements of a modern training curriculum. This study highlights the need to create an improved, competency-based curriculum that ensures the training of a new generation of surgeons, taking into account the growing evidence of the effectiveness of state-of-the-art training modalities such as simulation or proficiency-based training.

**Supplementary Information:**

The online version contains supplementary material available at 10.1186/s12909-024-05209-4.

## Background

With the widespread introduction of minimally invasive techniques in abdominal surgery, the demands on surgical residency training have changed significantly in recent decades. Teaching hospitals increasingly face the challenge of providing efficient and focused surgical training that takes into account the increasing demands on future surgeons as well as changing socioeconomic factors.

While laparoscopy offers a range of benefits to the patient, the surgeon faces numerous technical difficulties in performing minimally invasive procedures [1]: eye-hand coordination within a three-dimensional scene observed on a two-dimensional screen; attenuated haptic perception and feedback; fulcrum effect that leads to a scaling of the movement; limited freedom of manipulation; and suboptimal ergonomics. The significantly increased complication rates in the early days compared to open surgery highlight these difficulties. For example, during the advent of laparoscopic cholecystectomy as a standard procedure, there was up to a fivefold increase in bile duct injuries [2, 3].

Furthermore, socioeconomic factors have a fundamental influence on continuing education. In Switzerland, working hours are limited to 48 h per week, including mandatory learning time. However, the administrative workload has increased dramatically [4], and the compatibility of family and career as well as an increased demand for work-life balance are becoming more relevant. The increasing cost pressure in the Swiss healthcare system also requires efficient continuing education of residents. Finally, the shortage of physicians is an increasing problem [5, 6].

Teaching operative skills outside the operating room dates back to the 1980s. There is an abundance of evidence that simulation training is an effective way of acquiring laparoscopic skills in a safe and controlled environment without posing a threat to patient safety [7–9]. However, the integration of simulation training into surgical curricula is still controversial, and no surgical curriculum incorporates a fixed proportion of simulator training [10].

Another important change in surgical training is the transition from a volume-based Halsted approach of “see one, do one, teach one” [11] to a proficiency-based approach [12]. The goal is no longer to perform a certain number of operations but to achieve an objectively validated level of expertise and the ability to skillfully perform a procedure regardless of how many repetitions of the procedure were needed.

Despite the aforementioned advantages of simulation and proficiency-based training, their widespread acceptance and implementation in national surgical curricula is still lacking. Switzerland is no exception, as the evaluation of the official curriculum for surgical training shows [13]. Supplement 1 summarizes the requirements to complete surgical training in Switzerland. The minimum duration of training is 6 years. Training must be completed at a minimum of two certified hospitals (Table 1). Additionally, trainees are required to meet several formal requirements and fulfil a predefined surgical catalog. The surgical catalog does not specify whether an intervention must be minimally invasive or conventional. The trainee needs to complete several mandatory courses. Two theoretical exams must be passed: a multiple-choice exam after the two-year common trunk (that includes questions on all surgical subspecialties) and a final oral exam with case discussions at the end of residency.

**Table 1:** Categories of surgical training facilities in Switzerland according to the SIWF

**Category**	**Duration**	**Minimum procedures/year**				
	Max. training time	At institution	Performed by residents			
**A** **incl. University A(U)**	4 years	> 2700	> 1500	- criteria from B1/2 & B3 - habilitated head of department - tertiary care trauma unit incl. shock room - research activity		
**B3**	3 years	> 2000	> 1000	- criteria from B1/2 - accredited intensive care unit - 24/7 surgical emergency department		
**B2**	2 years	> 1200	> 500	- primary and secondary surgical care - basis care emergency department - outpatient care - at least 4 h/week of structured education		
**B1**	1 year	> 800	> 300			

The surgical training landscape in Switzerland has changed over the past years. Laparoscopy courses are offered by private companies (e.g., IRCAD [14]), hospital networks (e.g., OSTZ [15]) and foundations (e.g., Foundation for Gastrointestinal Surgery, "DavosCourse" [16]). In addition, there is an increasing network formation among hospitals to optimize surgical training. The Swiss College of Surgeons has begun to implement assessments in surgical education, but they are still in the development phase. These include “Direct Observation of Procedural Skills” (Surgical DOPS) and “Entrustable Professional Activities” (EPAs) [17].

The aim of this survey was to create a representative overview of the surgical training landscape in Switzerland and to obtain answers to the following questions:

1. How do postgraduate teaching hospitals cope with the mentioned challenges?
2. How is the gap between the existing curriculum and the challenges closed?
3. What are the needs of teaching hospitals for a further developed surgical curriculum?

## Material and methods

We conducted an online survey among all accredited surgical training centers in Switzerland based on the official list of the Swiss Institute for Continuing Education and Training SIWF/ISFM [18]. Hospitals that represented only surgical subspecialties or did not offer laparoscopy were excluded. Hospitals with more than one site were only considered once.

An online survey was created using the Survey Monkey tool (SurveyMonkey Inc., San Mateo, USA). The survey was conducted between 02/11/2022 and 30/12/2022. Both the design of the survey and the strategies to improve the response rate followed previously established rules [19–22]. The questionnaire was sent to each surgical head of a department.

All methods were carried out in accordance with relevant guidelines and regulations. We obtained written informed consent from all study participants (head of departments). Data collection was completely anonymized. Swiss Ethics Committee "Swissethics.ch" grants a general waiver for the use of purely anonymized data and an application with approval is not requested (Swiss Federal Act on “Research involving Human Beings 810.30”).

The survey was divided into 6 thematic areas with a total of 40 questions. Switzerland has four official languages: German, French, Italian and Rhaeto-Romanic. The questionnaire was drafted in German and translated to French using an online translation tool (DeepL SE, Köln, Germany). The French translation was validated by a bilingual person, and the survey was sent out in both German and French. The English transcription of the questionnaire (Supplement 2) is attached to this article as a supplement.

In the first part, general information about the training institution was collected. The second part addressed general surgical training issues, such as the possibility of completing the full surgical training for board certification or the time required to complete the full surgical curriculum. The following two parts focused specifically on laparoscopic training, with questions about the availability and type of simulators at each hospital and particular interest in high-fidelity and virtual reality simulators. The fifth part aimed to determine the aspects of the surgical curriculum. The last part had the most subjective character and included an assessment of the way the training path to board certification should be organized in the future. Data analysis was conducted using Microsoft Excel 365 (Microsoft Corporation, Redmond, USA).

## Results

We included 86 of 88 possible residency sites. Two centers that do not perform any laparoscopies were excluded (an emergency department and a traumatology center). All other heads of the surgical departments were contacted. Table 2 summarizes the most important key figures of the included training centers. The overall response rates were 56% (48/86) and 86% (19/22) for category A (U) and A hospitals, respectively.

**Table 2:** Key figures/indicators

Training centers			Residents				
**Hospital category**	**Included centers**	**Survey response** **(-rate)**	**Overall**	**Included residents in survey**	**Residency training in surgery**	**Board certification per year**	**Ratio specialist per resident**
Data source:	SIWF 2022^a^	Survey	SIWF 2022	Survey	Survey	Survey	Survey
**Total**	**86**	**48 (56%)**	**1020**	**627 (61%)**	**334 (53%)**	**75 (22%)**	**3:2 (1.5)**
A(U)	5	5 (100%)	106	106 (100%)	76 (72%)	21 (28%)	6:5 (1.2)
A	17	14 (82%)	330	285 (86%)	160 (56%)	31 (19%)	7:5 (1.4)
B3	20	10 (50%)	284	104 (37%)	44 (42%)	12 (27%)	9:5 (1.8)
B2	32	16 (50%)	246	121 (49%)	50 (41%)	11 (22%)	17:10 (1.7)
B1	12	3 (21%)	54	14 (26%)	4 (29%)	0 (0%)	3:1 (3.0)

^a^Official SIWF data 2022: Overview on residency training in Switzerland, https://www.siwf.ch/files/pdf28/2022_fg.pdf

A total of 627 residents are working at the responding departments, of whom 334 (53%) are in further training to become specialists in surgery. Category A (U) and A centers have a higher proportion of surgical training residents than category B hospitals (236 of 391 (60%) vs. 98 of 239 (41%), respectively). Seventy-five (22%) residents completed their residency training each year. The mean duration of surgical training to board certification is 6.5 years (SD ± 0.79).

### Educational network formation

Two-thirds of the centers (32) are cooperating in training networks. Of these, 27 (84%) created a surgical training curriculum in addition to the SIWF *(“Schweizerisches Institut für Weiter- und Fortbildung”*, Swiss Institute for Continuing Education and Training), with a focus on laparoscopic surgery. Twenty-six (81%) heads of department reported that their educational network offers complete residency training, including all rotations to other sites.

**Fig. 1:**
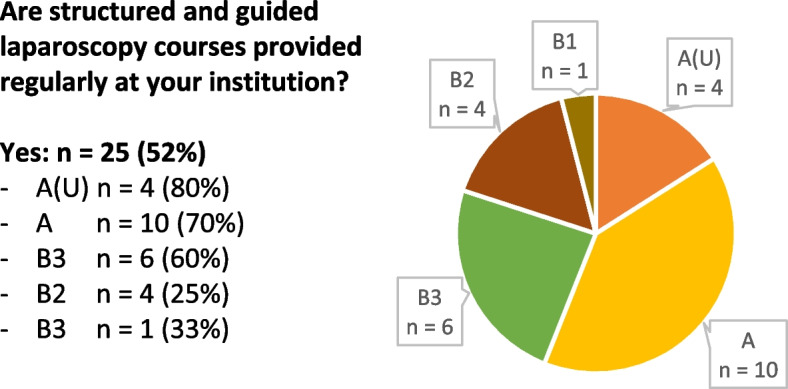
In-house laparoscopy courses

In-house laparoscopy courses are offered periodically at 25 (52%) hospitals, mainly in category “A” (Fig. 1). They focus mostly on basic laparoscopic skills.

### Self-training

**Fig. 2:**
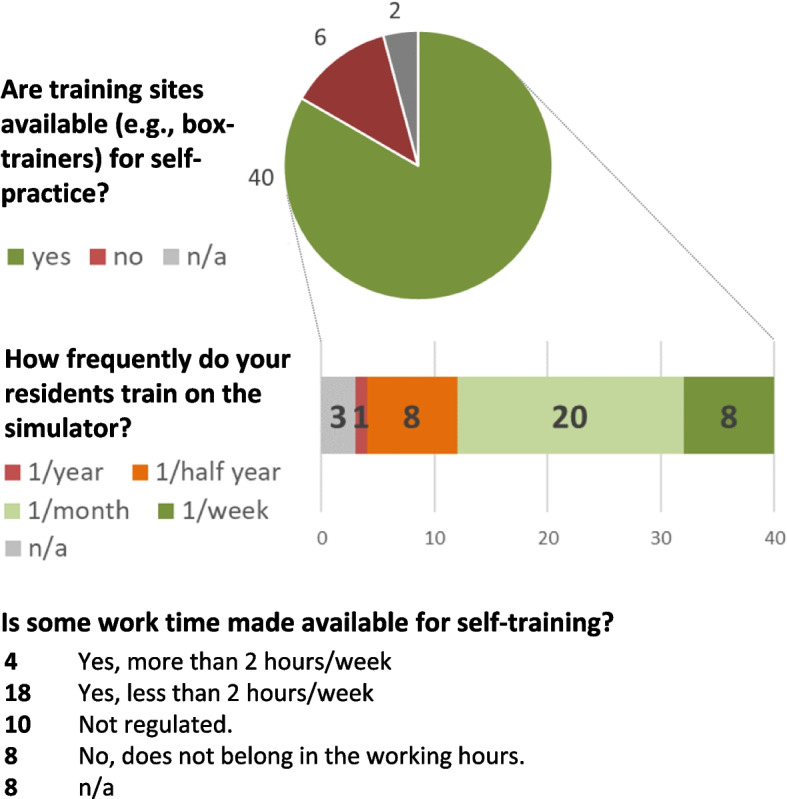
Self-training

Self-training sites are available at 40 (83%) hospitals (Fig. 2). In addition to commercial (27) and homemade (7) box trainers, high-fidelity trainers are available in 16 (33%) hospitals. Twenty-one (44%) of the heads of departments believe that high-fidelity trainers are superior to box trainers. The main reasons for the lack of self-training opportunities are high costs (2), lack of necessity (2) and lack of interest of the residents (4). Self-training sites are used infrequently by residents, although 2 or more working hours are made available by 22 institutions.

### Training curriculum and assessment

There is a mandatory training curriculum in 7 (15%) institutions; in 10, there is a defined practice plan for some specific skills, and in another 10, some unstructured exercises are available. Fifteen institutions (31%) offer a structured assessment of the training. However, assessments vary greatly, ranging from mandatory completion to tutor-based one-to-one teaching on the simulator. In 25 (52%) centers, manual skills are assessed only during the mandatory annual evaluation interview.

### Surgical curriculum and its further development

**Fig. 3:**
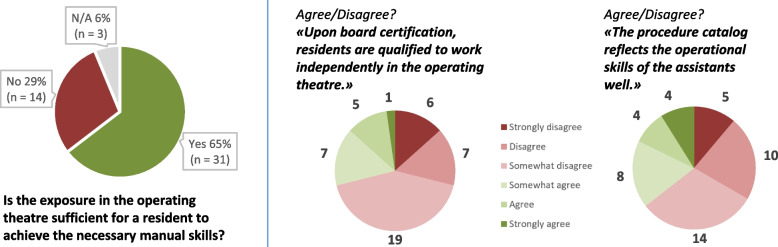
Surgical exposure

Department chairs of 31 hospitals (65%) indicate that surgical exposure in the OR is sufficient to acquire the necessary manual skills (Fig. 3). However, the question of whether residents are qualified to work independently as surgeons once they have obtained their board certification is viewed critically, and the majority of 71% (32) reject this, at least in part. In addition, it is clearly stated that the surgical catalog does not adequately reflect the manual skills needed to be proficient as an attending surgeon (64%).

**Fig. 4:**
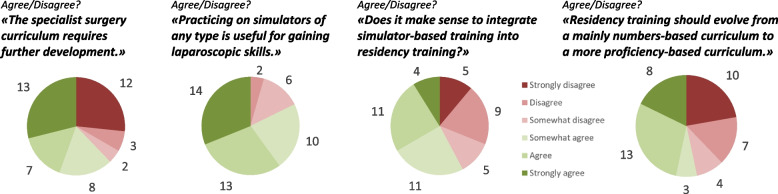
Future development of the surgical curriculum

The majority of the heads of departments agree with the statements that simulator training has a proven benefit for the acquisition of laparoscopic skills and that it makes sense to integrate this training into continuing education (Fig. 4). There is a trend toward restructuring the training curriculum from a number-based to a performance-based curriculum (53%) and establishing tools to assess the manual skills of residents (56%).

## Discussion

This is the first comprehensive survey to assess the surgical education landscape in Switzerland with a focus on laparoscopic surgery. The overall response rate of 56% is comparable to the response rates in a recently published systematic review by Banning et al. [23] on surveys in surgical education (59.4% for e-mail surveys).

If we consider only the large training centers (category A), the response rate rises to a remarkable 86%. The high response rate at these hospitals reflects the high value placed on surgical education. The comparatively low response rate of smaller hospitals is likely due to a different teaching attitude rather than to a lack of continuing education culture. Because smaller hospitals are only accredited for 1–2 years of training, there is less need to offer a structured continuing education program. Due to the small number of residents, training is likely organized in the sense of a one-to-one apprenticeship model.

### Interpreting the survey results

To combine the advantages of a small hospital with those of a large center, the data show that the majority of hospitals have formed educational networks. Small hospitals benefit from simplified recruitment and already well-trained residents, whereas large centers rely on smaller hospitals to provide their residents with complete residency training and to keep them in the company. However, smaller hospitals provide invaluable support on the path to becoming a competent surgeon. They offer the opportunity to take on a higher degree of responsibility and develop greater autonomy at an earlier stage of training [24, 25].

Due to the complexity of laparoscopic surgeries, the need for training facilities in addition to the operating room has been widely accepted. Simulation training allows training in a controlled, artificial environment where errors have no consequences other than providing the trainee with valuable experience [26]. Moreover, there is plenty of evidence that the main requirement for the rationality of simulation is met, namely, the transferability of skills from the simulator to the real operating room [27–29].

In particular, larger hospitals have recognized this fact and offer regular in-house laparoscopy courses (52%). These are mainly limited to basic skills. Smaller hospitals possibly lack the resources needed (material and teachers) as well as enough trainees; therefore, such courses are offered irregularly and cannot provide continuous training.

Similar to the situation of professional athletes and aviation, repetitive training of simple procedures is crucial to improve the performance of surgeons. Self-training (83%) is widely available in Swiss training hospitals. This seems to be the optimal solution to provide continuing training in surgery. These workplaces are cost-effective and preserve resources. There are several commercial products available and even models that can be created on one's own, even for those who are not "do it yourself" experts. Structured assessment of self-training results is indispensable. However, only 15% of hospitals offer a structured curriculum on the box trainer or simulator. The lack of assessment and curriculum is one of the most important reasons why self-training opportunities are used infrequently or not at all (Fig. 2). In addition, little to no work time is specifically allocated for self-training. The fact that these training opportunities are not used is independent of the type of simulator. Even high-fidelity trainers are not used without the presence of a mandatory curriculum.

The Swiss Surgical Curriculum currently does provide specifications with regard to the attainment of target figures but not the assessment of quality. Accordingly, a qualitative assessment of the technical skills of resident surgeons has thus far been left to individual institutions and is handled with varying degrees of consistency.

### Thoughts on creating a surgical curriculum

A future curriculum must recognize and account for the paradigm shift from the volume-based Halsted concept “see one, do one, teach one” [30] to a proficiency-based approach [12, 31]. The goal should no longer be a given number of operations but an objectively validated level of expertise and ability to proficiently perform a procedure independent of how many repetitions of the procedure were needed to achieve it.

Upon completion of surgical training, trainees should have a comparable level of expertise and be able to perform their tasks reliably and successfully. A goal-directed approach that aims to achieve a grade of expertise is far more reliable than depending on a case log alone [32]. The mere number of procedures conducted cannot be equated with mastery and is only a poor surrogate at best [33]. Making proficiency the primary requirement for board certification would emphasize the individualization of training, which has proven to be cost effective and appropriate in many cases.

When people’s lives or health are at stake, mistakes resulting from a lack of experience cannot be accepted. It is also ethically impermissible to subject patients to less than optimal therapy because it is administered by a person who does not have the required level of experience [34]. The ascending arm of the learning curve should not be completed on the patient, and before a resident begins operating on human beings, he or she should be properly trained. Simulation training is an option to circumvent the dilemma posed by the need to ensure continuity of care, which can only be provided by the next generation of surgeons.

Simulation training raises the question of the associated costs. However, the financial aspect is put into perspective when the potential benefits of simulation are considered. Automatically generated feedback saves human resources by eliminating the need for training under human supervision. Moving training from the operating room to the skills lab can save precious operating room time, the cost of which is approximately $36 to $37 per minute [35]. Teaching in the operating room prolongs the duration of procedures and thus the expense. It has been estimated that operations in which residents are trained last 12.6 min longer than cases in which faculty operate alone [36].

What merits comment is also the motivation factor of high-fidelity simulators, which makes trainees more likely to continue their practice on a regular basis. This is considered the most efficient training mode and is superior to occasional exercises at wider time intervals even with longer training sessions [37, 38]. The reason for this could be the gamified nature of the training, which turns the practice into an entertainment program [39].

The next important issue in this context is the structuration of training. The simulator alone is insufficient. It is only an object that must be integrated into a complex and carefully developed curriculum, including specific exercises, clear goals, structured feedback and validated means of assessing the outcomes. A number of assessment tools have been developed and validated [17, 40–42]. Whichever tool is chosen, it should meet the prerequisite of transparency and reproducibility and provide constructive feedback that aids goal-directed improvement (Table 3).

**Table 3:** Essentials of a modern surgical curriculum

Proficiency based
Individualization
Simulation
High fidelity
Practice on regular basis
Feedback
Assessment
Cost efficiency
Patient ‘s safety
Unification

### Limitations

First, a survey always delivers the subjective opinions of the addressees, which do not necessarily reflect the true conditions. It also cannot be guaranteed that the surveyed heads of departments were abreast of educational issues in their clinic. Not infrequently, there are specifically delegated team members who tend to these matters, and a request to forward the survey to the most suitable person was not explicitly included in the letter to the chiefs of departments.

Second, the response rate shows heterogeneity regarding the hospitals’ categories, with underrepresentation of the smaller training facilities.

Third, the survey design and content may be somewhat biased toward advocacy for simulation and state-of-the-art learning methods, which could have been negatively perceived by the participants or could have subconsciously directed their answers toward a positive assessment of simulation.

Finally, the survey was only conducted among the heads of departments and not among the residents or other professional groups (e.g. scrub nurses) involved in continuing education. The perspective of residents should be analyzed in a further survey in order to address any discrepancies in perspective.

Despite the aforementioned limitations, we believe that this study delivers important information and reflections for the development of future surgical curricula.

## Conclusions

This study is the first comprehensive evaluation of the surgical education landscape in Switzerland with a focus on laparoscopic training. The study highlights the gap between the accessibility and utilization of simulation training. It also emphasizes the need to establish an improved, structured curriculum. Neither governmental nor society-based regulations require simulation training for board certification. The financial reimbursement for surgical training is insufficient, as evidenced by the fact that there is no distinction between senior surgeons and residents in remuneration for hospital services. This is likely to result in a decreased number of surgical procedures performed by residents because of increasing commercial interest in surgical procedures. We strongly believe it is indispensable for surgical societies to increase their commitment to provide the missing link between research and the implementation of educational tools in structured education programs in the future.

### Supplementary Information

**Supplementary Material 1.**

**Supplementary Material 2.** 

## Data Availability

The datasets used and analyzed during the current study are available from the corresponding author on reasonable request. The questionnaire is available as supplemental material (Supplement 2).

## Abbreviations

OR: Operating room
SIWF: Schweizerische Institut für Weiter- und Fortbildung
ATLS: Advanced trauma life support
OSTZ: Ostschweizer Training- und Schulungszentrum (“Eastern Swiss Surgical Training Center”)
DOPS: Direct Observation of Procedural Skills
EPA: Entrustable professional activities
A(U): Training hospital category A in university
