# Supplementary material for: There is a need for a paradigm shift in laparoscopic surgical training: results of a nationwide survey among teaching hospitals in Switzerland
Source: BMC Med Educ. 2024 Feb 27;24:205. doi: 10.1186/s12909-024-05209-4 (PMC10900659; doi:10.1186/s12909-024-05209-4)
Supplement: Supplementary file 1 — Supplementary Material 1. [file 12909_2024_5209_MOESM1_ESM.docx]

| Supplement 1 - Swiss Surgical Curriculum according to SIWF | | |
| --- | --- | --- |
| Description of the surgical specialty | - both conservative and operative treatment of the surgical diseases and injuries - attention to the patient as a holistic human being with medical, social, ethical and economical sequels of his or her disease | |
| Goals | *Surgery related* | |
|  | - autonomous and responsible treatment of the surgical affections - ability to appropriate setting indication for operations and skillful carrying out surgical procedures as well as ensure the postoperative care - profound knowledge of anatomy, physiology, pathophysiology, pathology, diagnostic and pharmacology | |
|  | *Not surgery related* | |
|  | communicating and social skills, self-assessment, ability to introspection, constructive self-criticism, giving and receiving feedback | |
| Duration and structure | at least 6 years (or longer depending on fulfilling the requirements, especially case log) | |
|  | 2 years | 4 years |
|  | common trunk (general surgery, basics relevant for each surgical subspecialty) | subject specific |
|  | *Specific requirements* | |
|  | clinical surgery | 45 – 69 months: including 6 months of surgical of interdisciplinary emergency department |
|  | anesthesia or intensive medicine | 3-6 months |
|  | training facility category A | 2 years |
|  | training facility category B | 2 years |
|  | optional: surgical subspecialties (vascular-, thorax-, hearth-, hand-, plastic surgery, orthopedics etc.) | up to 2 years |
|  | optional: activity on university or research institute | maximal 2 years |
|  | activity on the very same hospital | maximal 4 years |
| Documentation | online platform for documentations of achieved learning goals and case log by each trainee with evaluation at least every year or on the occasion of the changing of the workplace | |
| Courses and Congresses | annually congress of the Swiss Surgical Society | At least 3 |
|  | acknowledged education events (including interdisciplinary tumor boards, lectures, on hand workshops) | At least 4 |
|  | SSC acknowledged two-day course (ATLS, Fixateur externe, radiation protection, ultrasound, preparatory seminar for board exam, AO trauma, The Davos Course – open and laparoscopic gastrointestinal surgery) | At least 2 |
| Publication and research activity | at least one publication as the first author in peer review journal or dissertation | |
| Surgical disciplines | Proficiency level | Basics |
|  | general and visceral surgery, traumatology | urology, orthopedic, pediatric surgery, oncology, thorax surgery, vascular surgery, hand surgery, plastic, reconstructive and aesthetic surgery |
| Operations` catalogue (case log) | emergency surgery | 85 procedures |
|  | general surgery + visceral surgery | 260 +165 procedures respectively including the beneath mentioned: |
|  | laparoscopy/laparotomy | 70 procedures |
|  | abdominal wall hernias | 65 procedures |
|  | stomach surgery | 7 procedures |
|  | appendectomy | 30 procedures |
|  | cholecystectomy | 30 procedures |
|  | colorectal | 10 procedures |
|  | hepatobiliary (excluding cholecystectomy) | 5 procedures |
|  | endocrine surgery | 10 procedures |
|  | proctology | 55 procedures |
|  | splenectomy | 3 procedures |
|  | stoma, small bowel | 50 procedures |
|  | Should the trainee carry out the sub-steps of the more complex procedure or should he or she assist to another resident, it can be credited to the case log (maximal two sub-steps/procedure) | |
| Certification | written exam (Basis exam) at completion of the common trunk: multiple choice test | Binary assessment with «pass» or «non-pass» |
|  | oral exam (final examination): case discussion (two cases from each: visceral surgery, traumatology and surgical subspecialties) |  |
