# Supplementary material for: There is a need for a paradigm shift in laparoscopic surgical training: results of a nationwide survey among teaching hospitals in Switzerland
Source: BMC Med Educ. 2024 Feb 27;24:205. doi: 10.1186/s12909-024-05209-4 (PMC10900659; doi:10.1186/s12909-024-05209-4)
Supplement: Supplementary file 2 — Supplementary Material 2. [file 12909_2024_5209_MOESM2_ESM.docx]

**Supplement 2: English translation of the questionnaire**

| 1 | Key figures of the training centre | *Questions about the training centre* |
| --- | --- | --- |
| 1.1 | Name of the training centre?  (The naming of the training centre enables us to inquire in case of ambiguities) | - Open question |
| 1.2 | Category of the training centre? | - A at the University - A - B1 - B2 - B3 |
| 1.3 | Number of employed assistants/residents? | - Open question |
| 1.4 | How many residents are in training to become a board certificated surgeon?  (Module independent) | - Open question |
| 1.5 | How many residents complete their residency training per year? | - Open question |
| 1.6 | Number of employed specialist title holders (up to chief level) | - Open question |
| 2 | **Surgical Education** | *General questions about surgical education* |
| 2.1 | Is your clinic affiliated with a surgical education network (e.g., hospital network, rotational positions, etc.)? | - Yes/No |
| *2.11* | If yes, which one? | - Open question |
| *2.12* | Does your education network have a structured internal education curriculum in addition to the SIWF requirements? | - Yes/No |
| *2.13* | Can the complete specialist training be completed in your training network? | - Yes/No/No disclosures |
| *2.14* | If so, what is the average number of years the residents need to complete their training and acquire the board certification? | - Open question |
| 2.2 | Are structured laparoscopy courses offered regularly at your clinic?  (with tutors) | - Yes/No/No disclosures |
| *2.21* | If yes, what type of courses? | - Open question |
| 2.3 | Is the exposure in the operating room sufficient for an assistant to achieve the necessary level of proficiency regarding manual skills? | - Yes/No/No disclosures |
| 3 | **Training in the laparoscopic surgery** | *Specific questions about laparoscopic training in and out of the operating room* |
| 3.1 | Are there permanently available laparoscopic training stations at your hospital where residents can practice independently? | - Yes/No |
| *3.11* | If yes, which ones? | Multiple choice:  - Commercial Box Trainer (e.g. Pelvi Trainer, etc.)  - Self-Made Box Trainers  - High Fidelity / Virtual Reality Simulators (e.g. Virtamed, Simbionics, SurgicalScience, etc.)  - Other (please specify) |
| *3.12* | In the event that training opportunities are available, how often are they voluntarily used by residents? | Single selection  - Regular (approx. 1/week)  - Occasional (approx. 1/month)  - Sporadically (approx. 1/half year)  - Rarely (approx. 1/year)  - Never |
| *3.13* | If there are no training opportunities, what is the reason for it? | Multiple choice  - Too high costs  - Lack of interest on the part of assistants, no need  - Lack of evidence for effectiveness of simulators  - Lack of need - there is enough practice during "real" operations  - Other (please specify) |
| 4 | **High Fidelity / Virtual Reality Trainer** | *Specific questions regarding the use of high fidelity / virtual reality simulators in advanced surgical training.*  *Answer questions 4.1 & 4.2 only if such a simulator is available in your clinic. Otherwise, proceed directly to question 4.3.* |
| 4.1 | If a high fidelity / virtual reality / computer simulator is available at your clinic, is it used more frequently than traditional training options? | - Yes / No / No simulator |
| *4.11* | If so, are there any reasons for this? | Multiple choice  - More fun (gamification)  - More realistic than Box Trainer  - Direct feedback from the simulator spurs on (score)  - Other (please specify) |
| 4.2 | Is the high fidelity / virtual reality simulator part of a clinic's internal education curriculum? | - Yes/No/No disclosures |
| 4.3 | Do you think it makes sense to include a high fidelity / virtual reality simulator as an integral part of a continuing education curriculum? | - Yes/No/No disclosures |
| 4.4 | Do the high acquisition costs (100,000 Swiss francs +) justify the purchase of a high fidelity / virtual reality simulator? | - Yes/No/No disclosures |
| *4.41* | Reason for the above answer (regardless of whether yes or no) | Multiple choice  Contra  - Acquisition too expensive  - There are more efficient ways of further education  Pro  - More efficient training  - The savings of a tutor makes up for the purchase cost  - Other (please specify) |
| 4.5 | Is a high fidelity / virtual reality simulator superior to a conventional simulator (e.g. box trainer)? | - Yes/No/No disclosures |
| 4.6 | If such a simulator were mandatory for official surgical training imposed by SIWF, would you purchase one? | - Yes/No/No disclosures |
| 5 | **Training Curriculum** | *What follow are questions about the use of a simulator in surgical training at your hospital. The type of simulator does not matter (box trainer, high fidelity / virtual reality trainer, etc.).* |
| 5.1 | What are the general conditions for independent training at your clinic? | Multiple choice  - Everyone does it on his or her own  - There are some prepared and described practice sequences  - There is a concrete practice plan (curriculum) for certain skills (e.g. preparation, suturing, camera work)  - There is a curriculum that must be completed on a mandatory basis  - Other (please specify) |
| *5.11* | What should such framework conditions ideally be like? | - Open question |
| 5.2 | Is training on the simulator actively integrated into the surgical training in your clinic? | - Yes/No/No disclosures |
| 5.3 | Is part of the working time made available for simulation training? | Single selection  - Yes, more than 2 hours/week  - Yes, less than 2 hours/week  - No, is part of free time  - Other (please specify) |
| 5.4 | Is there an in-clinic structured assessment on the simulator? | - Yes/No/No disclosures |
| 5.5 | In general, how are the manual skills of the residents checked? | Multiple choice  - On the occasion of the annual evaluations prescribed by SIWF.  - On the basis of the operation catalogue in the logbook  - During the periodic meetings of the management physicians, which take place specifically for this purpose  - On the basis of structured assessments in the operating theatre  - On the basis of structured assessments on the simulator  - No assessment is performed  - Other (please specify) |
| 5.6 | Do you consider practicing on simulators (any type) useful for learning laparoscopic skills? | Simple selection  1= no benefit, 6=maximum benefit |
| 6 | **Surgical Curriculum for board certification** | Statements |
| 6.1 | Comment on the following statements: |  |
| *6.11* | Upon obtaining the title of specialist, assistants are qualified to practice surgery independently. | 1 = absolutely agree, 6= disagree |
| *6.12* | The specialist surgery curriculum requires further development | 1 = absolutely agree, 6= disagree |
| *6.13* | The "operation catalogue" reflects the operational skills of the assistants well | 1 = absolutely agree, 6= disagree |
| *6.14* | Residency training should evolve from a purely numbers-based curriculum (operations` numbers, years of training, number of courses, etc.) to a more performance-based curriculum (based on skills). | 1 = absolutely agree, 6= disagree |
| *6.15* | Do you think it makes sense to integrate simulator-based training into residency programs? | 1 = absolutely agree, 6= disagree |
| *6.16* | "See one, do one, teach one" - this credo is outdated and does not belong in a modern continuing education curriculum | 1 = absolutely agree, 6= disagree |
| *6.17* | The establishment of evaluation methods of manual skills of assistants is desirable.. | 1 = absolutely agree, 6= disagree |
